# Multitargeted Herbal Prescription So Shiho Tang: A Scoping Review on Biomarkers for the Evaluation of Therapeutic Effects

**DOI:** 10.3390/ph16101371

**Published:** 2023-09-27

**Authors:** Nguyen Khoi Song Tran, Ji Hwan Lee, Myong Jin Lee, Jun Yeon Park, Ki Sung Kang

**Affiliations:** 1 College of Korean Medicine, Gachon University, Seongnam 13120, Republic of Korea; kauri87@gachon.ac.kr (N.K.S.T.); kleert26@gmail.com (J.H.L.); myongene@naver.com (M.J.L.); 2 Department of Food Science and Biotechnology, Kyonggi University, Suwon 16227, Republic of Korea

**Keywords:** So Shiho Tang, chronic inflammation, systemic, herbal medicine, multitarget, biomarker

## Abstract

Alternative medicines, especially herbal remedies, have been employed to treat infections and metabolism-related chronic inflammation because their safety and multidimensional therapeutic potential outweigh those of synthetic drugs. So Shiho Tang (SSHT), a well-known Oriental prescription (Xiao Chai Hu Tang in Chinese) composed of seven herbs, is traditionally prescribed to treat various viral infections and chronic metabolic disorders in Asia with or without the support of other natural medicines. To provide a general background on how SSHT is used as a medicinal alternative, we conducted a scoping review using the PubMed database system. Among the 453 articles, 76 studies used aqueous extracts of SSHT alone. This result included seven clinical studies and 69 basic studies: cell-based, animal-based, and ex vivo studies. The in vitro and clinical reports mainly focus on hepatic infection and hepatocarcinoma, and the documentation of in vivo tests of SSHT presents a wide range of effects on cancer, fibrosis, inflammation, and several metabolic disorder symptoms. Additionally, among the seven clinical records, two reverse-effect case studies were reported in middle-aged patients. In brief, this systematic review provides general knowledge on the natural remedy SSHT and its potential in phytotherapeutic primary health care.

## 1. Introduction

Abnormal biological functions of certain organelles can lead to many pathological conditions. As a common pathological condition, chronic inflammation is a therapeutic target for diseases, including infectious diseases, cancers, fibrosis, certain metabolic disorders, and vice versa [1,2,3]. Immune responses to external infections or dramatic internal metabolic shifts depend on various mechanisms [2,4]. Specifically, inflammatory cytokines, such as interleukin 2 (IL-2), interferon γ (IFN-γ), and tumor necrosis factor α (TNF-α) secreted by T helper cell 1 (Th1), participate in type 1 inflammation in response to cellular stimuli. Th2 cells secrete IL-4, IL-5, IL-10, IL-13, and tumor growth factor β (TGFβ) in type 2 immunity in response to body allergies and extracellular stimuli [5,6]. Therefore, etiopathology is a premise for chronic disease prevention and treatment.

A multidimensional medicinal approach has been successfully implemented using herbal medicines [7,8]. There are two major advantages of natural-origin therapies as alternatives: they are multitarget and naturally affordable. Unintended effects are the most observed concern in the pharmaceutical industry. Severe side effects can result in abnormal metabolism, permanent damage, and lethality [9]. For instance, antimicrobial studies have demonstrated that antibiotic resistance is the first consideration for pathogenic and anti-inflammatory drugs [10]. Some anti-allergy drugs, such as antihistamines, decongestants, antileukotriene agents, and corticosteroids, have been shown to lead to undesirable outcomes, including arrhythmia, vomiting, anxiety, neuropsychiatric disorders, nasal dryness, and irritation [11,12,13,14,15]. Thus, herbal remedies have been employed as substitutes and complementary medicines along with long-term synthetic drug medications. Traditional Chinese medicine (TCM) also plays a critical role in the complementary treatment of certain infectious diseases and cancers [16]. Phytotherapy has been developed to determine the appropriate use of multitargeted TCM, which can promote advantages and prevent possible risks [17].

So Shiho Tang (SSHT), or Xiao Chai Hu Tang in Chinese, has been an Oriental herbal medicine famous for fever, stomatitis, and gastrointestinal disorders for centuries in Asia [18]. In Japan, SSHT is well known as Sho-saiko-to or TJ-9, a chronic hepatitis medication [19]. SSHT is a mixture of different amounts of Radix bupleuri (Chaihu), Radix scutellariae (Huangqin), Rhizoma pinelliae (Banxia), Rhizoma zingiberis recens (Shengjiang), Fructus jujubae (Dazao), licorice (Gancao), and ginseng radix (Renshen) [20,21]. SSHT was evaluated as safe at doses of up to 2000 mg/kg in rat models [22,23] and 9000 mg/day in human participants aged 14–18 years [18]. According to a review by Kim et al., SSHT significantly lowered the elevated liver fibrosis markers and collagen types in fibrosis/cirrhosis-induced animal’s serum [24]. Aqueous STT has been reported to prevent and attenuate several chronic diseases, including chronic inflammatory skin diseases and gastrointestinal disorders [18,21,25]. The variation in the phytochemical features of SSHT limits the identification of the specific effects of each element, leading to challenging and incompatible approval processes in Western countries. Despite these concerns, SSHT has been successfully applied in numerous therapeutic prescriptions, including clinical trials in Asia [26,27]. The traditional East Asian medicine (TEAM) system has developed a basic mechanism by which TCM and SSHT work synergistically to boost medicinal efficacy and alleviate the reverse effects [28,29]. Although a recent bibliometric analysis summarized the application of randomized control trials of SSHT and Western medicine in examination designs [30], an outline of how SSHT alone is potentially participating in modern medicine has not been presented.

As long as TCM, particularly SSHT, is composed of many components, systems pharmacology will allow researchers to access multiple mechanisms of action without isolating a single compound [31,32]. Understanding the mechanisms of how drugs and the body interact at system levels is a key aspect of medications in general. In this study, we aimed to classify the medicinal applications of SSHT in cellular, preclinical, and clinical studies. Herein, we address two points: (1) indications of multitargeted biomarkers and significant dosages, and (2) suggestions for effective preclinical studies and mechanisms for subsequent clinical examinations of SSHT. Through this methodical approach, researchers can comprehend the mode of action of SSHT on various multitargeted biomarkers and signaling pathways in certain disease models as well as patients. Because valuable in vitro and in vivo records guarantee successful clinical outcomes, the novelty and limitations of these studies can be collected, analyzed, and applied to further examinations using systemic pharmacology analysis. This scoping review provides general knowledge on the use of SSHT and an interdisciplinary perspective to therapy.

## 2. Literature Research Strategy

**Figure 1:**
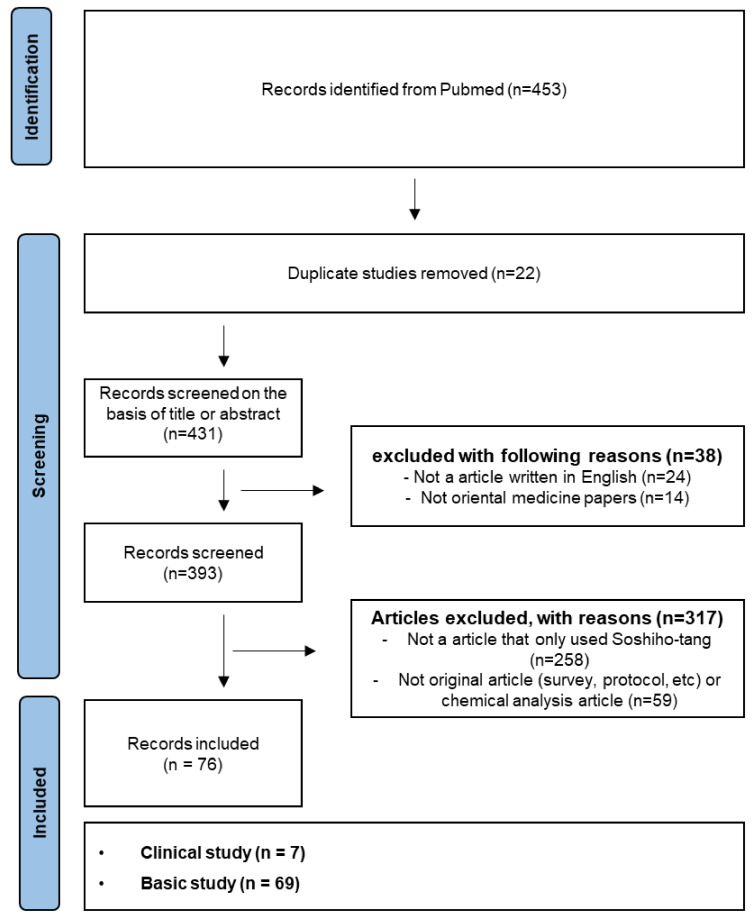
Flow chart of study selection indicates the retrieval process.

This review introduces the current status of cell-based, animal-based, and clinical studies on target diseases and the efficacy of SSHT. We used a search strategy to collect, identify, and classify literature on the applied diseases and the effects of SSHT on each disease. Considering that SSHT prescription is known by various scientific names, the following search terms were set through related reviews and databases: “soshiho-tang”, “so-shi-ho-tang”, “soshiho”, “sosiho-tang”, “so-si-ho-tang”, “sosiho”, “xiaochaihu”, “xiao-chai-hu-tang”, “xiaochaihu-tang”, “Xiao-chai-hu-decoction”, “Xiaochaihu-decoction”, “Xiaochaihudecoction”, “shosaikoto”, “shosaiko-to”, “sho-saiko-to” and “sho-sai-ko-to”. A literature search was conducted for articles published until 20 December 2022 on the PubMed database only. The literature was identified and classified according to the following inclusion criteria: (1) Oriental medicine articles, (2) written in English, (3) on the efficacy of SSHT alone or/with treatment, and (4) original articles (e.g., survey, protocol). The exclusion criterion was studies that performed only chemical analysis. In addition, data were extracted from predetermined items in the literature (e.g., types of and several herbs included in prescription, clinical, or basic studies; cell type or animal type in basic studies; disease-inducing method; details of intervention; extraction method; concentration (dose); and outcome measures) and reviewed with others (Figure 1). Three researchers independently reviewed and evaluated the eligibility of the titles, abstracts, and full texts of the articles. In cases of disagreement during the literature selection and data extraction processes, they discussed the implications. The data extracted after the war are summarized and presented in the table, along with narrative descriptions.

A total of 453 articles were retrieved using this search strategy. Literature that fulfilled the exclusion criteria was classified into cell, animal, and clinical studies by assessing the abstract and full text of the thesis according to the conditions stated. As a result of this process, 76 papers were included in the systematic review (Figure 1). The 76 results were divided into seven clinical studies and 69 basic studies. Regarding the efficacy of SSHT in basic research, 33 cell-based studies, 33 animal-based studies, and three in vitro and in vivo studies have been reported.

## 3. Results

### 3.1. In Vitro Studies on SSHT

Our analysis showed that one third (33.3%) of in vitro articles were published in Japan, followed by 22.2% in Korea, 11.1% in China, and 2.7% in Taiwan and the United States (Table 1). As shown in Table 1, SSHT is water-extracted and named Sho-saiko-to, So Shiho Tang, or Xiao Chai Hu, depending on the country. Among these publications, seven demonstrated anticancer effects, mainly in hepatocellular carcinoma [33]. Additionally, SSHT was reported to cure various cancer types, such as ovarian cancer [34], melanoma, colon cancer, and adenocarcinoma. Growth inhibition and cytotoxicity against cancer cells were examined using SSHT treatment. Zhao et al. evaluated not only the antiproliferative effect but also the apoptotic effect [35]. This study found that SSHT induced apoptosis through the expression of Bax, Bcl-2, CDK4, and cyclin-D1. Liu et al. examined the apoptotic effects of SSHT in a murine malignant melanoma cell line (Mel-ret) [36]. SSHT downregulates the expression of Fas-L, cdk4, cdk6, and pRB. Yamashiki et al. used peripheral blood mononuclear cells from patients with hepatocellular carcinoma and type C liver cirrhosis, and demonstrated that SSHT induced the dose-dependent production of tumor necrosis factor-α (TNF-α) and granulocyte colony-stimulating factor (G-CSF) [37]. Mizushima et al. examined the cytotoxic effects of different concentrations of SSHT (0.5, 1, 1.5, 2, and 2.5 mg/mL) [38]. Except for SBC-5 cells, all tested cell lines were cytotoxic to SSHT in a dose-dependent manner. Shao et al. reported no effect of SSHT on the viability of HCT116 and LOVO cells [39]. 

Seven studies have investigated the immunomodulatory activity of SSHT. Kawakita et al. demonstrated that SSHT is an IFN inducer for the peroral treatment of spleen cells [40]. The immunological status induced by the inflammatory factors reflects an imbalance between CD4+ and CD8+ T cells. Modulation of the CD4/CD8 ratio by SSHT has been observed in hepatic mononuclear cells but not in splenocytes [41,42]. The induction of cytokines, such as interleukin-1β (IL-1β), TNF-α, and granulocyte colony-stimulating factor (G-CSF), are peculiar effects of SSHT [43]. Another study investigated the levels of cytokine mRNA of IL-12, IL-1β, IL-10, TNF-α, G-CSF, and IFN-γ; they were upregulated by SSHT using the Gel doc system [44]. Matsumoto et al. investigated the histamine release and intracellular Ca^2+^ reactions in mast cells after SSHT [45]. Because mast cells play a key role in IgE-mediated allergic reactions, these findings indicate that they have an active allergic effect. SSHT has been studied for the production of prostaglandin E2 (PGE2), leukotriene B4 (LTB4), and superoxide in polymorphonuclear cells of individuals infected with the human immunodeficiency virus [46]. Data showed that PGE2 and superoxide decreased and LTB4 increased after SSHT treatment, suggesting that SSHT suppressed human immunodeficiency virus activity and, more precisely, modulated the immune reaction.

The in vitro protection effects of SSHT on other diseases have also been reported lately. Sakaguchi et al. reported the effects of SSHT on the treatment of chronic hepatitis [47]. They verified the cytotoxic protection of the treated macrophage cell line J774A.1 by using SSHT. TNF-α production also showed significant inhibitory effects in cells treated with SSHT. Understanding that periodontal disease leads to inflammation of the gingiva, Ara et al. found the anti-inflammatory potential of SSHT in human gingival fibroblasts (HGFs) with LPS as an inducer [48]. The results suggest that SSHT inhibited PGE2 and cyclooxygenase2 (COX2). Two other studies have reported the anti-inflammatory effects of SSHT by using RAW264.7 cells, an LPS inducer and immunomodulator, and dexamethasone as a positive control. Oh et al. elicited the effect of SSHT on LPS-induced macrophages through the NF-ĸB and MAPK pathways [49]. They concluded that SSHT inhibited nitric oxide, immune response cytokines, and NF-ĸB through the dephosphorylation of the MAPK pathway in LPS-induced RAW264.7 cells. Choi et al. also employed RAW 264.7 cells in LPS-induced model, and found a similar inhibition of pro-inflammatory cytokines, such as IL-6 and TNF-α, via pretreatment or co-treatment with SSHT [50]. In a study of acute pancreatitis, Zhan et al. used LPS-stimulated rat pancreatic AR42J cells, and SSHT played an important role in the treatment of acute pancreatitis by acting on the key genes of MAPK3, IL-6, and TP53, which were associated with inflammation [51]. 

**Table 1:** Summary of designs and biomarkers of in vitro studies on SSHT.

Study Design	Cell Type	Inducer	Extract Type	Treatment (* Effective Concentrations)	Positive Control	Biomarker & Outcome	Authors	Country
Antitumor effect	Mel-ret cell		Aq	400 *, 2000 µg/mL		Fas-L, cdk4, cdk6, pRb	Liu et al. (1998) [36]	Japan
Antitumor effect (Proliferation)	HCC cell line (KIM-1), cholangiocarcinoma cell line (KMC-1)		Aq	400, 1000 *, 2000 *, 10,000 µg/mL		Morphological change, DNA synthesis	Yano et al. (1994) [33]	Japan
Antitumor effect (Cytotoxic effect)	PLC, JH, HuH-7, HepG2, HepG3, H69, H69/CDDP, SBC-4, SBC-5, PC-10, ABC-1, LCD, LCT-8		Aq	0.5, 1, 1.5, 2.0, 2.5 mg/mL		Cell viability	Mizushima et al. (1995) [38]	Japan
Immune response (Induction of cytokines)	Peripheral blood mononuclear cell (PBMC)		Aq	3.1 *, 12.5 *, 50 *, 200 * µg/mL		TNF-α, G-CSF	Yamashiki et al. (1996) [37]	Japan
Anticancer effect (Proliferation)	Ovarian cancer cell (KF-1, MN-1, A2780, KF-r, MN-r, A2780cp)		Aq	25, 50, 100, 200, 500, 1000 *, 5000 * µg/mL		Annexin V-FITC	Zhu et al. (2005) [34]	Japan
Antitumor effect (Apoptosis)	Human hepatoma cell line (Huh7)		Aq	0.5 *, 1.0 *, 1.5 * mg/mL		Bcl-2, Bax, cyclin-D1, CDK4	Zhao et al. (2017) [35]	China
Anticancer effect (cancer comorbid with depression)	HCT116 & Lovo		Aq	10, 20, 40, 80, 180, 320 μg/mL		Cell viability	Shao et al. (2021) [39]	China
Immune response (Induction of interferon)	Spleen cell	IFN-a/β	Aq	7.5, 15, 30, 60, 120, 250, 500 μg/mL		IFN	Kawakita et al. (1990) [40]	Japan
Immune response (Inductions of cytokines)	Peripheral blood mononuclear cells (PBMNC)		Aq	200 µg/mL		IL-1β, TNF-α, G-CSF	Yamashiki et al. (1996) [43]	Japan
Immune response	Peripheral blood mononuclear cells & polymorphonuclear cells (PMNC)		Aq	25, 50 *, 100 * μg/mL		PGE2, LTB4, superoxide	Miyamoto et al. (1996) [46]	USA
Immune response	Mast cell	DNP-As	Aq	0.01, 0.1, 1 *, 10 * mg/mL		Histamine release, Ca^2+^ response	Matsumoto et al. (1998) [45]	Japan
Immune response (Cytokine mRNA expression)	Human peripheral lymphocyte	LPS	Aq	200 μg/mL		IL-12, IL-1β, IL-10, TNF-α, G-CSF, IFN-γ	Huang et al. (2001) [44]	Japan
Immune response (Proliferation of T cell subsets)	Hepatic mononuclear cells, splenocyte	Anti-CD3 mAb	Aq	62.5 *, 125 *, 250 * µg/mL		CD4/CD8	Ohtake et al. (2005) [41]	Japan
Immune response	Splenocyte, CD4 T cell		Aq	10 *, 25 *, 50 *, 100 * μg/mL		IL-4, IFN-γ	Kang et al. (2009) [42]	Korea
Immune response	J774A.1 cell	LPS	Aq	10 *, 20 *, 50 * µg/mL		Endotoxin-induced cytotoxicity (TNF-α)	Sakaguchi et al. (2004) [47]	Japan
Anti-inflammatory effects	HGFs		Aq	0.1, 0.3 *, 1 * mg/mL		PGE2, COX-2	Ara et al. (2008) [48]	Japan
Anti-inflammatory effects	RAW 264.7	LPS	Aq	10 *, 50 *, 100 * μg/mL	Dexamethasone	TNF-α, IL-6, Nitric Oxide, NF-κB, IκBα, ERK, p38, JNK	Oh et al. (2013) [49]	Korea
Anti-inflammatory effects	RAW 264.7	LPS	Aq	1, 10, 100, 250 *, 500 *, 1000 * μg/mL	Dexamethasone	IL-6, TNF-α, IFN-γ	Choi et al. (2021) [50]	Korea
Anti-inflammatory effects (Acute pancreatitis)	AR42J cell	LPS	Aq	12.5, 25 *, 50 *, 100 * μM		IL-6, IL-1β, TNF-α, MAPK3, TP53	Zhan et al. (2021) [51]	China
Immune response (Chronic HBV infection)	PBMC		Aq	50 *, 100 *, 300 * μg/mL		IFN-γ, anti-HBe & anti-HBc production	Kakumu et al. (1990) [52]	Japan
Liver diseases (Chronic active hepatitis B and C)	Peripheral blood mononuclear cells, polymorphonuclear cells (PMNC)		Aq	100 μg/mL		IL-10	Yamashiki et al. (1997) [53]	Japan
Liver diseases (Chronic hepatitis B)	HepG2.2.15 cell		Aq	10 *, 20 *%(vol/vol)		JAK2, STAT3, HBsAg	Chen et al. (2017) [54]	China
Liver diseases	Hepatocyte		Aq	3 *, 30 *, 300 * μg/mL		iNOS, nitric oxide	Hattori et al. (1995) [55]	Japan
Lipid metabolism	Hepatocyte	20% lipid emulsion	Aq	50, 100 *, 200 *, 400 *, 800 μg/mL		G6PD, ME, FAS, ATGL, LPL, PPARα	Zou et al. (2019) [56]	China
Liver fibrosis	Hepatic stellate cell (HSCs)		Aq	10, 100, 250, 500 *, 1000 * μg/mL		Type I and type III procollagen	Kayano et al. (1998) [57]	Japan
Liver fibrosis	Rat hepatic stellate cells (HSCs)		Aq	10, 100 *, 500 *, 1000 * µg/mL		MMP-13, MMP-2, TIMP-1, TIMP-2	Sakaida et al. (2004) [58]	Japan
Aging (Autocrine growth of human keratinocyte)	Normal human keratinocyte		Aq	100 *, 500 * µg/mL		IL-1α	Matsumoto et al. (1997) [59]	Japan
Atopic dermatitis (AD) symptoms	HaCaT cell	TNF-α/IFN-γ	Aq	10 *, 20, 50 *, 100 *, 500 μg/mL		ICAM-1, HO-1, NF-κB, Nrf2	Lee et al. (2019) [21]	Korea
Aging (UVB-induced skin damage and photoaging)	HaCaT cell	UVB irradiation	Aq	10 *, 50 *, 100 * µg/mL		MMP-1, MMP-9	Im et al. (2020) [60]	Korea
Mitogenic activity	Spleen cell	LPS		0.1, 1 *, 10 *, 100 * µg/mL		Mitogenic response ([^3^H] thymidine uptake)	Hiroko et al. (1987) [61]	Japan
Antiviral activity	Human neonatal foreskin fibroblast cell line (CCFS-1/KMC)		Aq	25 *, 50 *, 100 *, 200 * µg/mL	Ribavirin	IFN-α, IFN-β, CVB1	Cheng et al. (2006) [62]	Taiwan
Anti-inflammatory effects (Calprotectin expression)	Human oral epithelial cell (TR146)		Aq	10, 25 *, 50 *, 100, 250 µg/mL		S100A8, S100A9, Calprotectin, ADM, AZU1, CAMP, CST3, DEFB1, DEFB4, DEFB103A, LCN2, IL-1α, IL-6, TNF-α G-CSF, MUC5B, IL-1R1	Hiroshima et al. (2010) [63]	Japan
Antithrombotic effect	Platelet	Collagen, thrombin, AA, ADP	Aq	200 *, 400 *, 800 * μg/mL	ASA	Serotonin, TXB2	Lee et al. (2013) [64]	Korea
Antiobesity effect	3T3-L1		Aq	50 *, 100 *, 200 * µg/mL	GW9662	PPAR-γ and C/EBP-α, FAS, perilipin, FABP4, triglyceride, leptin	Yoo et al. (2016) [65]	Korea
Immune response (mRNA & microRNA expression)	Mouse primary hepatocyte		Aq	500 µg/mL		P450 metabolism, cell cycle pathway, PPAR pathway, MAPK pathway	Song et al. (2014) [66]	Korea

Aq, Aqueous; eNOS, endothelial nitric oxide synthase; iNOS, inducible nitric oxide synthase; COX2, cyclooxygenase 2; MMPs, matrix metalloproteinase. adrenomedullin; ADM, azurocidin 1; AZU1, cathelicidin; CAMP, cystatin C; CST3, β-defensin 1; DEFB1, b-defensin 2; DEFB4, b-defensin 3; DEFB103A, lipocalin 2; LCN2, interleukin-1α; IL-1α, interleukin-6; IL-6, tumor necrosis factor α; TNF-α, granulocyte colony-stimulating factor; G-CSF, mucin 5; MUC5B, interleukin-1a receptor; IL-1R1, thymus-independent; TI, arachidonic acid; AA, acetylsalicylic acid; ASA, Thromboxane B2; TXB2. (*) concentration with significant effect. Accordingly, SSHT and quercetin were associated with anti-inflammation and blocked not only IL-6, TNF-α, and IL-1β but also MAPK3 and TP53.

Liver-related studies on SSHT have been divided into two categories: hepatitis virus [19,52,53,54] and liver disease (non-virus) [55,56,57,58]. The efficacy of SSHT was investigated in peripheral blood mononuclear cells from patients with chronic hepatitis. SSHT was found to enhance IFN-γ and alleviate HBe and HBc production dose-dependently. Moreover, SSHT induced IL-10, CSF, and TNF-α. Chen et al. treated HepG2 cells with 10% or 20% mXCHD-containing serum and found that the expression of hepatitis B surface antigen (HBsAg) decreased with 10% and 20% mXCHD [54]. The mRNA and protein expression of STAT3 increased in the group treated with 20% mXCHD serum, and no difference was observed in the expression level of JAK2. Zou et al. [56] tested the mRNA expression of adipogenesis markers (G6PD, DGAT2, and ME1) and lipolysis-related genes (ATGL, PPARα, and LPL) in 20% lipid emulsion (LE)-induced hepatocytes. SSHT treatment reverses LE-induced lipid accumulation and improves cell viability. Hatorri et al. studied the effect of SSHT on NO biosynthesis and found that SSHT dose-dependently induced NO production and iNOS mRNA expression in the presence of IFN [55]. Kayano et al. and Sakaida et al. clarified the inhibitory effect of SSHT on the formation of stellate cells isolated from male Wistar rats [57,58]. However, Kayano et al. reported that SSHT in 500 and 1000 µg/mL doses inhibited the transcription level of type I and III procollagen in scar tissue. Sakaida et al. demonstrated the upregulation of MMP2 and MMP13 and the downregulation of TIMP1, TIMP2, and IL-1α, which have an important function in immune regulatory and inflammatory responses were produced in keratinocytes treated with SSHT [59]. These extracts also regulate the proliferation of cultured human epidermal keratinocytes. Atopic dermatitis (AD) is a commonly observed skin condition. Lee et al. investigated the effect of SSHT on AD-like symptoms in TNF-ĸ/IFN-ĸ-activated human keratinocytes (HaCaT) and found that SSHT treatment increased the expression of heme oxygenase -1 (HO-1) and Nrf2 but decreased the expression of intercellular adhesion molecule-1 (I-CAM) and NF-ĸB [21]. Another dermatological study demonstrated protection against UVB-induced skin damage and photoaging after SSHT treatment [60]. SSHT, MMP1, and MMP9 were downregulated in UVB-irradiated HaCaT cells.

In addition, one study showed the mitogenic activity of SSHT by using murine splenic cells [61], and another study mentioned that SSHT neutralized Coxsackie B virus type 1 (CVB1)-induced cytopathic effects in foreskin fibroblast cells [62]. Another study reported the role of SSHT in calprotectin and AMP expression in oral epithelial cells [63]. Lee et al. examined the antithrombotic activity of SSHT [64], and Yoo et al. examined the antiobesity effects of SSHT [65]. In addition, Song et al. used microarray to analyze the correlation between temporally upregulated and downregulated patterns in primary mouse hepatocytes treated with SSHT [66]. A conclusion was drawn that the P450 metabolism, cell cycle, PPAR, and MAPK pathways are associated with the liver regenerative activity of SSHT. In Korea, Taiwan, and Japan, but not in China, antitumor examinations of SSHT have used methods associated with apoptosis detection, such as flow cytometry for annexin V and Western blot detection of Bcl-2 and Bax protein levels. Several immunity-related studies have been conducted on SSHT. Specifically, in most studies conducted in Korea, RAW 264.7 cells are employed as the target model, and LPS is used as an inflammatory inducer to show anti-inflammatory effects. Different concentrations of SSHT have been applied to various cell types to develop relevant methods and targets.

### 3.2. In Vivo Studies on SSHT

Cancer will be a leading cause of death in the 2020s, according to the WHO [67]. Aqueous extracts of SSHT cured cancer in ddY and BALB mouse models (Table 2). Drinking 2.5 g/kg/day of SSHT per month blocked cancer reproduction and metastasis, similar to that of IL-2 monotherapy. In addition, after tumor-transplanted mice drank 1600–2500 mg/kg SSHT for 2–4 weeks, their survival rate increased and their tumor size decreased [68,69]. At the lower dose of 500 mg/kg, oral administration showed antitumor activity by alleviating fibrinogen and glycogen levels in ddY mice after 5 d [70]. In addition to its action in cancers, SSHT has been demonstrated to have anti-fibrotic ability in rats fed up to 1% chow for a minimum of 8 weeks [71,72]. Sakaida et al. reported that 1% chow remarkably altered hydroxyproline and hyaluronic acid and reduced fibrotic markers, for example, COL3A1, myofibroblast-like cells, and glutathione S-transferase placental form (GST-P) positive foci, in induced rats compared with that of a choline-supplemented-amino acid-defined diet group [72]. Another study performed on BALB/c mice for 16 weeks confirmed that a very low dosage of SSHT (5 mL/kg/day) per os inhibits liver injury by decreasing scaring factor levels such as TGF-β1, Hsp47, α-SMA, Col1A1, and Col3A1 [73]. Moreover, the survival rate of ddY male mice with recombinant human tumor necrosis factor (rhTNF)-induced lethality recovered with 500 mg/kg/day feeding with SSHT [74].

In addition to fibrosis, other pathological features of chronic inflammatory diseases are downregulated when hosts are exposed to SSHT. Notably, studies have been conducted on the immune response in female mice because females have more efficient immune responses than males do [75,76]. In 1987, Kawakita et al. used C3H/He female mice as models for B-cell maturation and bacterial infection. SSHT was pre-injected intraperitoneally (I.P.) at different doses (100 and 250 mg/kg) before inflammatory induction. With 100 mg/kg I.P. of the SSHT extract, immune leukocyte levels were notably reduced after 6 h and 4 d of exposure [77]. With 250 mg/kg of the extract, the plaque-forming process was inhibited, and mature IgM+ IgD+ B cells were elevated on days 10 and 14 of treatment in response to exogenous stimulation [78]. I.P. injection of SSHT also rescued the renal failure of C3H/He and (BALB/c × DBA/2) F~(CDF) female mice [79] and showed significant anti-inflammatory activity at a low dose of 100 mg/mouse by increasing granulocyte macrophage colony-stimulating factor (GM-CSF) secretion [80]. In Nagatsu et al., prostaglandin E2 (PGE2) production decreased, and the membrane fluidity of murine host macrophages was successfully improved by SSHT in a dose- and time-dependent manner [81]. According to many reports, the oral administration of SSHT has been frequently used and has exhibited positive outcomes. In studies from 1994 and 1995 in Japan, SSHT aqueous extract was orally administered to C3H/He mice with chronic hepatitis at a high dose for 12–24 h and notably augmented natural killer (NK) cell activities [82,83]. At 500 mg/kg/day for 5 d, SSHT improved endotoxemia by altering lipid peroxide, xanthine oxidase, SOD, GPx, -tocopherol, and respiratory control index in male mice [84,85].

Recently, in ovalbumin-induced inflammation in BALB/c female mice, 3 weeks of feeding with 100–200 mg/kg/day SSHT decreased Th2-type cytokine levels while activating heme oxygenase-1 (HO-1) protein expression [86]. This data is consistent with the data from the positive control group treated with montelukast sodium, which effectively suppressed immunologic response stages in the muscles, lungs, and entire airway. In addition, an oral dosage of 50 and 100 mg/kg/day diminished IL 6, TNF-α, IL-1, and IFN-γ levels after 17 d [87]. Research in rats has also revealed the anti-inflammatory effects of SSHT. After treatment with 450 mg/kg/day, the aggregation of macrophages (granuloma) and certain lipids was alleviated compared with that of indomethacin, a granuloma inhibitor, and the control group in Wistar male rats [88,89]. The Cytochrome P450 (CYP P450) family of heme isozymes are responsible for drug biotransformation via oxidation. Hence, the CYP inducer rifampicin was used in a positive drug–drug interaction study. Another study by Li et al. performed in Sprague Dawley rats demonstrated the P450s-related drug–drug interaction effect of SSHT in dose-dependent trials in comparison with that of the rifampicin group [90].

**Table 2:** Summary of designs and biomarkers of in vivo studies (and 1 ex vivo study) on SSHT.

Target Study		Animal (Sex, Age, Body Weight)	Inducer	Type of Extracts	Administration (Frequency/Period)	Experimental Group	Positive Control	Biomarker & Outcome	Authors	Country
Lethality	Anti-lethality	ddY mice (male, 18–20 g)	rhTNF	Aq	500 * mg/kg/day (Oral, 1/day, day 2 & 6 of induced, 72 * h)	4 groups		Survival rate *	Sakaguchi S. et al. (1991) [74]	Japan
Cancer	Antitumor	ddY mice	Ehrlich tumors	Aq	1600 * mg/kg (Oral-drinking, 2 weeks)	10 groups		Survival rate *, TNF *, tumor weight	Haranaka K. et.al. (1985) [68]	Japan
Cancer	Antitumor activity, shock symptoms	ddY mice (male, 18–20 g)	LPS	Aq	500 * mg/kg/day (Oral, 1/day, 5 day)	4 groups		NO_2_, Fibrinogen *, Glycogen *	Sakaguchi S. et al. (1996) [70]	Japan
Cancer	Carcinoma	BALB/c mice (female)		Aq	2.5 * g/kg/day (Drink, 30 day)	5 groups	IL-2	IL-6 *, tumor weight *	Huang et al. (1997) [69]	Japan
Fibrosis	Hepatic foci	Sprague Dawley rats (male)	N-nitroso morpholine	Aq	0.5% *, 1% chow (Oral, 8 weeks)	3 groups		GGT *, GST-P * lesion staining, T lymphocyte*	Tatsuta et al. (1991) [71]	Japan
Fibrosis	Liver fibrosis	Wistar rats (male, 140–150 g)	Choline-deficient-amino acid-defined diet	Aq	1% * Chow (Oral, 16 weeks)	5 groups	Choline-supplemented-amino acid-defined diet	Hydroxyproline *, hyaluronic acid *, ALT, AST, COL3A1 *, myofibroblast-like cells *, GST-P lesion *	Isao et al. (1998) [72]	Japan
Fibrosis	Liver fibrosis	BALB/c mice (female, ~20 g)	S. *japonicum*	Aq	XCH-L: 5 * mL/kg/day, XCH-M: 15 * mL/kg/day, XCH-H: 30 * mL/kg/day (Oral, 16 weeks)	6 groups		Serum ALT *, AST *, ALP *, HA * & PIIINP *, ALB * & GLOB *, TGF-β1 *, Hsp47 *, α-SMA *, Col1A1 *, Col3A1 *	Huang et al. (2020) [73]	China
Inflammation	*P. aeruginosa* infection	ICR and C3H/He mice (female)	*P. aeruginosa*	Aq	100 mg/kg (I.P., pretreat 6 h * or 4 days *)	3 groups		Leukocytes *	Kawakita et al. (1987) [77]	Japan
Inflammation	Peritoneal macrophage	C3H/HeJ & (BALB/c × DBA/2)F~(CDF) mice (female)		Aq	3 * mg, 5 mg/mouse/day (I.P., 1 time, 4 days)	3 groups		Acid phosphatase & N-acetyl-/3-D-glucosaminidase	Kumazawa. et al. (1988) [79]	Japan
Inflammation	Immune response	ICR mice (male), CBA mice (female)		Aq	1.2 g/kg/day (Oral, 1, 2, 3 *, 5 * day)—0.24, 0.6 *, 1.2 * g/kg/day (Oral, 3 days)	4 groups		PGE2 *, antigens, ARA, membrane fluidity *	Nagatsu et al. (1989) [81]	Japan
Inflammation	Colony-stimulating factors	C3H/He mice, (female)	Carrageenan	Aq	100 * mg/kg (I.P., serum I.V.)	3 groups		GM-CSF	Yonekura et al. (1990) [80]	Japan
Inflammation	Ovalbumin-induced inflammation	BALB/c (OVA)-induced mice (female)		Aq	100 *, 200 * mg/kg/day (Oral, 1/day, 18–23 days)	5 groups	Montelukast	Th2-type cytokines *, eotaxin, (HO)-1 *	Jeon et al. (2015) [85]	Korea
Inflammation	Cachexia-related symptoms	BALB/c CT-26-bearing mice		Aq	50 * and 100 * mg/kg/day (Oral, 1/day, 17 days)	4 groups		IL-6, TNF-α, IL-1, IFN-γ	Kim et.al. (2016) [86]	Korea
Inflammation	Peyer’s patches (IgA production)	C3H/He mice (female)	LPS	Aq	200, 500, 1000 *, 2000 * mg/kg/day (Oral, 1/day, 2 day)	5 groups		SRBC-IgA & -HRBC IgA	Tauchi et al. (1993) [91]	Japan
Inflammation	Drug-drug interaction (CYPs)	Sprague Dawley Rats (male, 200–240 g)		Aq	Low dose, 1.7 * g/kg/day Medium dose, 3.4 * g/kg/day High dose, 6.8 * g/kg/day (Oral, 1/day, 3 & 6 days)	Control groups Treatment groups	CYP inducer (Rifampicin)	P450s (Cyp1a2, Cyp3a1, Cyp2d6, Cyp1b1)	Li et al. (2021) [89]	China
Inflammation	Endotoxemia	ddY mice (male, 18–20 g)	Endotoxin	Aq	500 * mg/kg/day (Oral, 1/day, 5 days)	4 groups		Lipid peroxide *, Xanthine oxidase *, SOD *, GPx *, α-Tocophero *, nonprotein SH *, acid phosphatase *, LDH *	Sakaguchi. et al. (1993) [84]	Japan
Inflammation	Granuloma	Wistar rats (male, ~200 g)	Carrageenin cotton pellet	Aq	450 * mg/kg/day (Oral, 1/day/8 days)	4 groups	Indomethacin	Granuloma weight *, acid-soluble glycoprotein *, sialic acid	Yoshida et al. (1993) [87]	Japan
Inflammation	NK activities	C3H/He mice (female)		Aq	500, 1000 * mg/kg (Oral 12 h * & 24 h)	3 groups		GM1, CD3, CD4, CD8, NK cell *	Kaneko et al. (1994) [82]	Japan
Inflammation	Granuloma	Wistar rats (male)	Carrageenin cotton pellet	Aq	450 * mg/kg/day (Oral, 1/day/8 days)	4 groups	Indomethacin	Vit E *, cholesterol, phospholipid, lipid peroxide *, granuloma weight *	Yoshida et al. (1994) [88]	Japan
Inflammation	Endotoxemia	ddY mice (male, 18–20 g)	Endotoxin	Aq	500 * mg/kg/day (Oral, 1/day, 5 days)	4 groups		[Ca^2+^] *, Mg^2+^, Ca^2+^-ATPase *, respiratory control index *	Sakaguchi et al. (1994) [84]	Japan
Inflammation	Macrophage function	Sprague Dawley rats (male, 150–155 g)	Gum arabic	Aq	1 g/kg/day (Oral, 3 weeks, 22 *–28 days)	4 groups		Superoxide anions	Fujiwara et al. (1995) [92]	Japan
Inflammation	NK activities	C3H/He mice (female)		Aq	1000 mg/kg, 1–12 * fraction (Oral, 1 time, 12 h)	12 groups		NK activities *	Yamaoka et al. (1995) [83]	Japan
Inflammation	B-cell maturation	C3H/He mice (female)	TNP-LPS (TI-1), TNP-Ficol (TI-2), SRCB (TD)	Aq	250 mg/kg (I.P., 4, 7, 10 *, 14 * days)	Control group Treatment group		Plaque-forming cells (PFC), IgM+ IgD+ * B cell, Thy1 antibody	Kawakita et al. (1987) [78]	Japan
Metabolic disorder	D-galactosamine -induced liver injury	ICR mice (male, 18–22 g)		Aq	Low dose 0.02 * g/kg/day Medium dose 1 * g/kg/day High dose 5 * g/kg/day (Oral, 1/day, 14 days)	6 groups	Biphenyl dicarboxylate	IL-6 *, TNF-α *, Fas*, Fas-L *, Bcl-2 *, Bax *	Zhou et al. (2012) [93]	China
Metabolic disorder	Perimenopausal disorder	Kunming Mice (female, 18–22 g)		Aq	2.3, 7 *, 21 * g/kg/day (Oral, 1/day, 8 weeks)	8 Groups	E2 & fluoxetine	HPA/HPO axis, Erβ, TPH2	Zhang et al. (2020) [94]	China
Metabolic disorder	Age-induced amnesia	Fischer F254 rats (male)		Aq	120 * mg/kg/day (Oral, 1/day, 72–110 * week-old)	4 groups	α-Tocopherol nicotinate	PAR failure *	Amagaya et al. (1990) [95]	Japan
Metabolic disorder	Liver injury	Wistar rats (male, 150–160 g)	D-galactosamine	Aq	1 * g/kg (Oral/I.P., 1 time, 24 h *)	8 groups		Albumin *, total protein *, 5′-nucleosidase *, glucose 6-phosphatase *, serum TG	Ohta et al. (1997) [96]	Japan
Metabolic disorder	Chronic pancreatitis	Wistar rats (male, 170–190 g)	Dibutyltin dichloride	Aq	10 * g/kg/day (Oral, 1/day, 28 days)	3 groups		Exocrine pancreatic function (PABA *), TGF-β1 *, TGFβRII *, Smad3 *, Smad7	Zhang et al. (2013) [97]	China
Metabolic disorder	Anti-hyperlipidemia & antiatherosclerosis	Hypercholesterolemic C57BL/6J mice (male, 20–24 g)		Aq	1.2 * g/kg/day (Oral, 1/day, 2–4 * weeks)	4 groups		Cholesterol *, T cell *, cholesterol oleate *, HDL, LDL, Acyl-CoA *, ACAT, NCEase *	Shen et al. (1996) [98]	Japan
Metabolic disorder	Hypercholesterolemia	ICR mice (male)		Aq	1.2 g/kg/day (Oral, 1/day, 2 weeks)	4 groups		PGE2 *, IL-1, NO *, LPC	Inoue et al. (1996) [90]	Japan
Metabolic disorder	Hypercholesterolemia	New Zealand White rabbits (male, 1.5–2 g)	LPS	Aq	3% chow (Oral, 20 * weeks)	3 groups		Monocyte *, cholesterol, LDL	Shen et al. (1996) [99]	Japan
Metabolic disorder	Tolbutamide bioavailability	Sprague Dawley rats (male, 302–376)		Aq	500 * mg/kg/day (Oral, 1/day, 6 days)	Single (2 groups) Multiple (2 groups)		Absorption rate & bioavailability of Tolbutamide	Nishimura et al. (1999) [100]	Japan
Metabolic disorder	Radical scavenging	Wistar rats (male)		Aq	500 * mg/kg (Oral, 1 time, plasma collection at 1, 2, 4, 6, 10, 12 *, 24 *h)	3 groups	α-Tocopherol & ascorbic acid	O_2_- *, -OH *, DPPH *,	Egashira et al. (1999) [101]	Japan
Metabolic disorder	Gastric function	Sprague Dawley rat (male, 215–347 g)		Aq	250 *, 750 * mg/kg (Oral, 1 time, 20 & 40 min)	Control group Treatment group		Gastric emptying rate (GER)	Nishimura et al. (2001) [102]	Japan
Metabolic disorder	Hepatic microvascular dysfunction	Wistar rats (male, 200–250 g)	Gut ischemia/reperfusion	Aq	1 * g/kg/day (I.G., 1/day, 7 days)	5 groups		Leukocytes * (pericentral * & midzonal region), NPS *, TNF-α *, ALT	Horie et al. (2001) [103]	Japan
Metabolic disorder	Tolbutamide permeability	Ex vivo, Sprague Dawley rats (male)		Aq	50 * mg/kg (I.P., 10–60 min)	Control group Treatment group		Epithelial membrane permeability of Tolbutamide	Nishimura et al. (2010 [104])	Japan

Aq, aqueous; I.P., intraperitoneal injection; TNF, tumor necrosis factor; PGE2, Prostaglandin E2; ARA, Arachidonic acid; I.V., intravenous; GM-CSF, Granulocyte macrophage colony-stimulating factor; PAR, passive avoidance responses; GGT, γ-glutamyl transpeptidase; GST-P, Placental glutathione S-transferase; HRBC-IgA, horse erythrocyte-IgA; SRBC-IgA, Sheep red blood cells-IgA; SOD, Superoxide dismutase; GPx, Glutathione peroxidase; nonprotein SH, nonprotein sulfhydryl; LDH, Lactate Dehydrogenase; NK, Natural Killer Cell; GM1, Ganglioside; CD, cluster of differentiation; Vit E, Vitamin E; ATPase, adenosine triphosphate catalyst; NO_2_, Nitrogen Dioxide; HDL, High Density Lipoprotein; LDL, low-density lipoprotein; ACAT, Acyl-CoA: cholesterol Acyltransferase; NCEase, neutral cholesterol ester hydrolase; IL-1, Interleukin-1; NO, Nitric oxide; LPC, Lysophosphatidylcholines; IL-2, Interleukin-2; DPPH, 2, 2-diphenyl-1-picrylhydrazyl; I.G., Intragastric; NPS, nonperfused sinusoids ; ALT, alanine aminotransferase; (HO)-1, enzyme heme oxygenase-1; IFN-γ, interferon gamma; Fas, Fas Cell Surface Death Receptor; FasL: Fas ligand; Bcl-2, B-cell lymphoma 2; Bax, bcl-2-like protein 4; HPA/HPO, hypothalamic-pituitary-adrenal/hypothalamic-pituitary-ovarian;, Erβ, Estrogen receptor beta; TPH2, Tryptophan hydroxylase 2; E2, estradiol; P450s, CYP, cytochrome p450; TGF-β1, transforming growth factor beta 1. (*) concentration with significant effect.

Furthermore, SSHT has been used to treat many metabolic disorders, including hepatic and pancreatic injuries, hyperlipidemia, and atherosclerosis. Experiments performed on male ICR mice and rabbits showed positive results for hypercholesterolemia reduction with low-dose absorption of SSHT (1.2 g/kg/day for 2 weeks) and 3% chow for 20 weeks [98,99]. Significance was analyzed in comparison with that of the biphenyl dicarboxylate-treated group, which was proven to abolish ALT in chronic liver disease models. Similarly, 1.2 g/kg/day of SSHT administered orally for 4 weeks initiated anti-hypercholesterolemia and antiatherosclerosis activity by attenuating cholesterol, T cells, cholesterol oleate, and acyl-CoA levels in hypercholesterolemic C57BL/6J mice [96]. In addition, hepatic injury in Wistar rats was rescued with 1 g/kg/day oral, I.P., and intragastric (I.G.) administration, which altered albumin, glucose 6-phosphatase, and leukocytes (pericentral and midzonal regions) [97,103]. By recovering exocrine pancreatic function (PABA) and dropping TGF-β1, TGFβRII, and the Smad3 protein level, SSHT was also verified as a chronic pancreatitis therapy in rats [97]. Different doses of SSHT were applied to mice with liver damage and perimenopausal disorder and had better results than the positive control groups did [93,94]. Studies by Nishimura et al. in Sprague Dawley rats have demonstrated the increased insulin sensitivity potential of oral and I.P.-administered SSHT by extending tolbutamide absorption [100,104]. Other reports in rats have shown that oral treatment with SSHT suppressed age-induced amnesia, scavenged radicals, and boosted the gastric emptying rate at modest doses [95,101,102]. These findings correlated with the positive treatment results of α-tocopherol, an antioxidant that scavenges lipid peroxyl radicals.

In summary, the aqueous extract of SSHT exhibits a wide range of therapeutic effects owing to its multicomponent characteristics. Among the 36 in vivo examinations of SSHT therapeutic ability, five were from China, two were from Korea, and the remainder were from Japan. Studies conducted in Japan were primarily performed in the 1990s and 2000s by using single- or double-dose treatments over multiple timeframes. By contrast, reports from China and Korea have been conducted in a dose-dependent manner over a single timeframe. Because fibrosis and inflammatory conditions have a wide range of effects on the body, various biomarkers have been enrolled, for example, lymphocyte accumulation, TNF-α, IL-6, and IL-1. By contrast, studies on cancer or metabolic disorders have focused on disease-specific markers such as tumor weight and tumor necrosis factor (TNF) expression in cancer research.

### 3.3. Clinical Studies on SSHT

Seven studies on SSHT were analyzed: four randomized controlled trials (RCTs) and three case reports (Table 3). For the efficacy evaluation of SSHT, four reports—one case of gastrointestinal disease in atopic patients, one case of depression in a patient with malignant cancer, and one case each of liver cirrhosis and hepatitis—were employed. In addition, two clinical cases reported that hepatotoxicity in middle-aged individuals could result from SSHT ingestion; one case successfully rescued liver function in a patient with chronic hepatitis C.

Four RCTs have evaluated the efficacy of SSHT on gastrointestinal diseases in patients with AD, depressive symptoms in patients with cancer, cirrhosis, and chronic active hepatitis. Sixty AD patients with gastrointestinal disorders [25] were enrolled and diagnosed using the Hanifin and Rajka criteria, with an AD (SCORAD) index score between 15 and 49. The participants were randomly assigned to the SSHT and placebo (PLA) groups at a 1:1 ratio, and efficacy was evaluated after 4 and 8 weeks. The participants were orally administered SSHT or PLA thrice daily for 4 weeks. This clinical study showed no statistically significant changes in AD severity or digestive symptoms in patients with AD treated with SSHT. However, as a complementary alternative treatment for AD, SSHT has been reported to alter steroid ointment dependence and improve the quality of life in patients with AD. A study on depressive symptoms in patients with cancer [39] was conducted to investigate the effects of SSHT on depression-associated tumors and its potential mechanisms. Specifically, a PLA-controlled trial was conducted in cancer patients with comorbid depressive symptoms to evaluate the effects of SSHT on depression scales, tumor-related immune indicators, and gut microbial composition. Seventy-two participants with lung, breast, colorectal, thyroid, and other cancers were randomly assigned to either the SSHT or PLA groups at a 1:1 ratio. The participants received 19 g of SSHT or PLA twice daily via oral administration for 6 weeks. Sixty-one participants completed the study. After 6 weeks of intervention, a significant decrease in the SDS score was observed in both groups. Antidepressant effects were more pronounced in the SSHT group than in the PLA group, as revealed by the differential SDS values (ΔSDS). The circulating cytokines assay further indicated that SSHT treatment downregulated TNF-α and IL-6 levels relative to the baseline and/or the PLA groups. The results of fecal 16S rRNA sequencing showed no significant differences between the Chao and Shannon indices on an operational taxonomic unit (OUT)-based principal component analysis (PCA).

A study on patients with cirrhosis [105] was conducted to evaluate the preventive effect of SSHT on hepatocellular carcinoma (HCC) development. Two hundred and sixty patients with cirrhosis were randomly assigned to two groups at a 1:1 ratio and matched for age, sex, presence of hepatitis B surface antigen, and severity of liver damage. Patients in the trial group were administered SSHT at a daily oral dose of 7.5 g in addition to the conventional drugs administered to the controls. The patients were prospectively monitored for 60 months, and the cumulative incidence of HCC and survival rates in the two groups were calculated. As a result, the 5-year survival curve of the trial group was higher than that of the control group, and for patients without HBs antigen, the difference was significant. Clinical research on chronic active hepatitis [106] enrolled 222 patients with the disease and conducted a double-blind, multicenter clinical study. One hundred and sixteen patients received SSHT at a daily oral dose of 5.4 g for 12 weeks, followed by the same dose for another 12 weeks. One hundred and six patients received PLA containing SSHT (0.5 g) for 12 weeks, followed by a crossover to SSHT for another 12 weeks. The medications assigned to the patients were coded by a controller and randomly assigned to the patients. This study showed that serum AST and ALT levels decreased significantly with the administration of SSHT. In addition, in patients with chronic active type B hepatitis, a tendency toward a decrease in HBeAg and an increase in anti-HBe antibodies was also observed.

Three case reports on the efficacy and adverse effects of SSHT have been published. In one case report on the efficacy of SSHT, 24 patients with chronic hepatitis C [107] were enrolled in a case study. The participants were orally administered medication thrice a day for 12 months. Liver function, hepatitis C virus (HCV) load, and liver biopsy histology were assessed before and after the intervention. Improvement in AST and ALT was observed in 16 (67%) and 18 (75%) participants, respectively. In nine subjects who showed improvement in their Knodell’s histology activity index (HAI) score, paired liver biopsies before and after treatment showed improvement. Case reports on the adverse effects and toxicity of SSHT were published in 1995 and 2006, respectively. The study in 1995 [108] reported the side effects of SSHT in four patients: a 51-, 52-, 58-, and 42-year-old woman; SSHT (7.5 g) was orally administered one or more times per day for 2 d up to 1.5 months. All patients who visited the hospital showed elevated levels of ALT and AST in their blood, and liver biopsies showed signs of hepatitis. Symptoms improved after the discontinuation of the drug. In a 2006 case report [109], a 52-year-old woman exhibited weakness, fatigue, and tea-colored urine after continuous consumption of SSHT twice daily for 1.5 months. Laboratory examination upon admission revealed elevated serum levels of aspartate aminotransferase (AST), alanine aminotransferase (ALT), γ-glutamyl transpeptidase, alkaline phosphatase, total bilirubin, and direct bilirubin. Liver biopsy revealed acute hepatocellular hepatitis symptoms. The symptoms improved after the discontinuation of the drug, and the liver biochemical tests normalized two months later.

**Table 3:** Summary of designs and biomarkers of clinical studies on SSHT.

Target Disease	Study Design (Sample Size, n)	Type of Preparation Form	Extraction Method	Dose	Duration (Frequency)	Control	Outcomes	Results	Authors	Country
Gastrointestinal disorders of AD patients	RCT (60: I 30, C 30)	NA	NA	NA	6 weeks (3/days)	PLA	(1) SCORAD index (2) Amount and frequency of ointment application for AD (3) Dermatology quality of life index (4) Safety evaluation	(1) (2) not statistically significant (3) reduced (4) normal range	Lee et al. (2021) [25]	Korea
Depressive symptoms of cancer patients	RCT (72: I 36, C 36)	Granule	Aq	9.5 g/pack	6 weeks (2/days)	PLA	(1) Depressive scales (SDS) (2) Circulating cytokines assay (3) Gut microbial composition	(1) *p* < 0.05 (2) *p* < 0.05 (TNF-α, IL-6) (3) not significant	Shao et al. (2021) [39]	China
Cirrhosis	RCT (260: I 130, C 130)	NA	NA	7.5 g/day	60 months (1/days)	PLA	(1) Cumulative incidence of HCC (2) Survival rate	(1) inhibitory effect (2) high survival rate	Oka et al. (1995) [105]	Japan
Chronic active hepatitis	RCT (222: I, C)	Granule	NA	5.4 g/day	24 weeks (3/days)	PLA	(1) ALT (2) AST (3) HBeAg	(1) (2) decreased blood levels (3) decreased HBeAg (increased anti-HBe antibody)	Hirayama et al. (1989) [106]	Japan
Hepatitis C	Case report (24)	Granule	Aq	2.5 g/pack	12 months (3/days)	-	(1) Liver function (2) HCV viral load (3) Liver biopsy histology	(1) improved (2) mixed (3) improved	Deng et al. (2011) [107]	USA
Adverse effect	Case report (1)	NA	Aq	NA	1.5 month (2/days)	-	(1) ALT, AST (2) Liver biopsy	(1) increased (2) revealed lesion	Hsu et al. (2006) [109]	Taiwan
Adverse effect	Case report (4)	Granule	NA	7.5 g/day	6–7 weeks (1/days)	-	(1) ALT, AST, ALP, γGT, bilirubin (2) Liver histology	(1) increased (2) revealed lesion	Itoh et al. (1995) [108]	Japan

AD, atopic dermatitis; ALT, alanine aminotransferase; AST, aspartate aminotransferase; HBeAg, hepatitis B e antigen; HCA, hepatitis C virus; HCC, hepatocellular carcinoma; SCORAD, atopic dermatitis score; APL, alkaline phosphatase; γGT, γ-glutamyl transpeptidase, PLA, placebo group; VAS, visual analog scale. NA, not applicable.

## 4. Discussion

**Figure 2:**
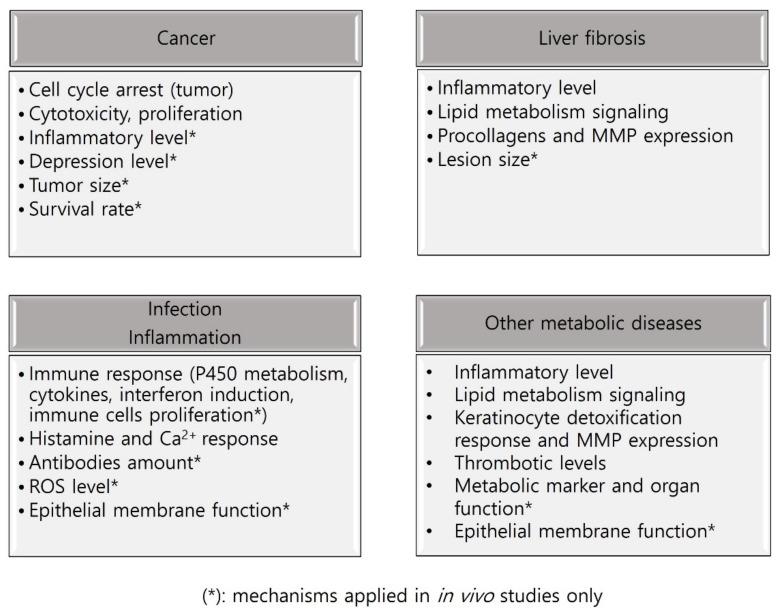
Target diseases and related mechanisms of SSHT in in vitro and in vivo experiments.

SSHT is a traditional herbal prescription used in East Asian medicine, particularly in Korea. In present study, we first provide an overall summary of previous in vitro, in vivo (Figure 2), and clinical research of SSHT effects on certain diseases and metabolic disorders. Subsequently, this will give the readers an insight into how multi-targeted biomarkers can be successfully engaged in TCM therapeutic studies.

In many cell-based studies (Table 1), hepatocytes and hepatocarcinoma cells have been treated with aqueous SSHT. SSHT is usually extracted using distilled water and freeze-dried before applying it to the experiments. Based on research data, SSHT was earlier assigned for various hepatic diseases medication including hepatitis virus infection and cancer alleviation. Therefore, various biomarkers were assessed in the SSHT-treated hepatic cells. As shown in the large category of immune reactions in blood cells, there is antithrombotic activity in platelets, endotoxin-induced cytotoxicity in J774A.1 cells, and histamine release in mast cells. Anti-inflammatory effects of SSHT were evaluated in HGFs, AR42J cells, and RAW264.7 cells. In addition, IL-6 is the only common biomarker of the anti-inflammatory effect of SSHT in the summarized articles. In addition, SSHT has been proven to protect keratinocytes and has antitumor activity in various cancer cells. In spleen cells, one study tested mitogenic activity, and another study tested the induction effect of interferon. Regarding the in vitro system, a wide range of cells have been utilized to determine the therapeutic potential of SSHT by examining many biomarkers using various methods.

Among the 36 studies performed on animal models (Table 2), 2.8% investigated the efficacy of SSHT on anti-lethality, 8.3% reported anticancer and antifibrosis effects, and 36% studied metabolic-related diseases. The largest proportion of in vivo examinations were anti-inflammatory studies, accounting for 44% of the total. Broad preclinical applications of the traditional SSHT remedy have been reported for decades. Owing to its multicomponent features, this herbal medicine exhibits a wide range of therapeutic effects, including antitumor, anti-lethal, antifibrosis, anti-inflammation, and antimetabolic activities. In general, oral administration has been employed for most case studies of SSHT and other folk remedies because many components can be distributed safely by the gastrointestinal system. Feeding a modest dosage of 100 mg/kg/day protected rodent models from certain infections after 2 weeks. At 500 mg/kg/day per os, SSHT recovered murine cancers and inflammatory endotoxemia after 5 d of treatment. For the long-term treatment of cancer and hepatic fibrosis, a low dosage of SSHT (5 mL/kg/day) can be applied for 8–16 weeks. In metabolic disease therapies, a high dosage of 1.2 g/kg/day has been used for anti-hypercholesterolemia and anti-atherosclerotic activities in mouse and rabbit models for 2–4 weeks. Additionally, I.P. injection induced significant curing effects of SSHT on inflammation and metabolic disorders in rodent models because large amounts of herbal extracts can be promptly absorbed through the peritoneum into the body. SSHT can be injected at 100–1000 mg for the recovery of anti-inflammatory and insulin resistance in mice and rats. On the other hand, animal studies have been controversial owing to ethical concerns, and conducting experiments on animals requires comprehensive knowledge and caution.

Seven clinical studies on SSHT have been reported: four RCTs and three case reports (Table 3). The four RCTs evaluated the efficacy of SSHT on gastrointestinal illness in patients with AD, depressive symptoms in patients with cancer, cirrhosis, and chronic active hepatitis. The efficacy of SSHT in patients with AD showed improvements in the SCORAD index, gastrointestinal symptoms, and atopic dermatitis symptoms. Based on these results, our expectation is that SSHT will reduce the dependence on steroid ointments and improve the quality of life of patients with AD. A study on depressive symptoms in patients with cancer showed progress in depression, tumor-related immune indicators, and gut microbial composition [39]. Another study on liver cirrhosis showed that SSHT initiated an improvement in the cumulative incidence of HCC and the survival rate in patients with liver cirrhosis [105]. This gives rise to the utilization of SSHT in chronic liver illnesses treatments. In addition, the publication of SSHT medicinal application on chronic active hepatitis showed a decrease in serum AST and ALT values and HBeAg, but an increase in anti-HBe antibodies in patients with chronic active type B hepatitis [106]. In three case reports, SSHT was effective in alleviating hepatitis C symptoms by improving liver lesion and function [107]. However, two clinical cases in elderly groups reported hepatotoxicity resulting in elevated blood ALT and AST levels and liver tissue damage after SSHT ingestion [108,109]. Of the clinical research on SSHT, efficacy as well as toxicity in the liver accounted for the largest portion (5 cases), followed by gastrointestinal diseases and depression. These results show that SSHT administration at appropriate doses should be taken into consideration, which can help recover not only the liver but also gastrointestinal diseases, AD, and depression.

**Figure 3:**
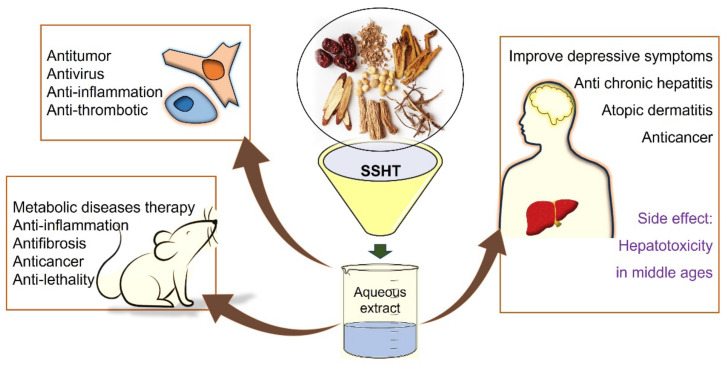
Schematic description of SSHT therapeutic application.

SSHT is a multitargeted herbal formula comprising various medicinal herbs with potential therapeutic effects. The evaluation of its effects involves identifying specific biomarkers, which are measurable indicators that can reflect changes or responses to the treatment (Figure 3). Since inflammation is a symbol of a wide range from acute to chronic diseases, inflammatory mediators were targeted for many medical studies. Herein, we have summarized the effective biomarkers which were enrolled in this review as follows.

(I) Inflammatory markers: SSHT modulated the inflammatory response by altering cytokines like interleukins (IL-1β, IL-4, IL-6, IL-10, IL-12), Th2, TNF-α, TGF-β, IFN-γ, and G-CSF. In addition, P450s and COX-2/PGE2 system also take part in drug metabolism and tumor genesis mediation, respectively. The modulation of these factors presented the therapeutic capacity of SSHT on inflammatory responses, acute and chronic disease, apoptosis, and cancers.

(II) Immune function markers: SSHT might influence immune function by evaluating markers such as leukocytes (CD3, CD4+, and CD8+ T-cell), granulocytes, and natural killer (NK) cell activity, as well as histamine, which could shed light on its immunomodulatory anti-allergy, anti-chronic disease (liver fibrosis), and anticancer effects.

(III) Oxidative markers: The herbal prescription’s antioxidative properties can be assessed by measuring biomarkers such as ROS, NO, iNOS, and endogenous antioxidant SOD and GPx levels. These markers can also be employed for inflammation state and apoptosis research.

(IV) Cell cycle progression and apoptosis markers: The inhibition of SSHT on certain cell cycle regulative biomarkers including Cyclin D, CDK (4 and 6), mitosis, and DNA synthesis levels could be targeted for cancers’ research. Additionally, apoptosis markers like TNF family (Fas-L) and Bcl2 are aims of cytotoxicity and apoptosis studies of SSHT.

(V) Cellular signaling markers: The herbal prescription’s multi-targeted effects may involve modulation of various cellular signaling pathways, such as MAPK (ERK, p38), NF-κB, IκBα, JAK-STAT, Nrf2/HO-1, and PI3K/AKT. Measuring the activity or expression levels of signaling molecules (FAS, ATGL, LPL, PPARs, MMPs, α-SMA, and certain indicative proteins) and transcription factors can provide insights into their mechanisms of action in fibrosis, metabolic disorders, and chronic diseases.

(VI) Metabolic markers: SSHT might have implications for metabolic health. Biomarkers such as blood glucose, insulin, vitamins, enzymes (AST/ALT, TPH2, ACAT, NCEase), hormones (HPA/HPO, Erβ), antigens and antibodies (SRBC-IgA and -HRBC IgA), collagen types, and lipid profiles (LDL, HDL, TG, and cholesterol) can be monitored. These metabolic markers were applied in fibrosis, inflammation state, cancer, acute and chronic disease, and infection research work.

(VII) Other biological indicators: The therapeutic effects of SSHT can be investigated by measuring granuloma weight (inflammations), tumor weight (cancers), fibrosis lesion level (fibrosis), respiratory index (endotoxemia), membrane fluidity (immune response), absorption rate (diabetes), SCORAD index (gastrointestinal disorders), gastric emptying rate (gut gastric function), depressive scale and gut microbial composition (mental diseases), and liver biopsy (liver diseases) as well.

SSHT represents the integration of traditional herbal knowledge with modern research, making it a potential candidate for complementary and alternative medicine. The multitargeted nature of the herbal prescription may offer a broad spectrum of effects, potentially addressing multiple aspects of a health condition simultaneously. As biomarker research advances, it might enable personalized treatment approaches with SSHT, tailoring the prescription based on individual patient profiles. Further research is necessary to establish the safety profile of SSHT and standardize its preparation to ensure consistent quality and efficacy. Rigorous clinical trials are essential to establish the therapeutic benefits of SSHT, providing evidence for its use in various health conditions.

It is important to note that scientific research in the field of herbal medicine is continually evolving, and more recent studies might have expanded our understanding of SSHT and its effects. For the most up-to-date and comprehensive information, it would be better to consult the latest scientific literature and consult with qualified healthcare professionals. Recently, efforts to understand the disease by integrating conventional molecular biological experiments, bio-big data analysis, and AI technologies have increased rapidly. In order to know the polypharmacological effects of multicomponents, a lot of time and effort is only required through conventional experiments [108,109]. Therefore, it is possible to develop a deeper understanding of the disease and better treatments through a more integrated approach.

Our work analyzed the literature on the protection of the traditional remedy SSHT in not only cell and animal-based tests but also in clinical models. We have summarized the mechanisms of certain multitargeted biomarkers involved in the conducted experiments and made a brief outline. Therefore, this review paper can give readers a comprehensive view of the SSHT therapeutic potential.

## Data Availability

The data presented in this study are available on request from the corresponding author.
